# Growth of White Leghorn Chicken Immune Organs after Long-Term Divergent Selection for High or Low Antibody Response to Sheep Red Blood Cells

**DOI:** 10.3390/ani14101487

**Published:** 2024-05-17

**Authors:** Christa F. Honaker, Robert L. Taylor, Frank W. Edens, Paul B. Siegel

**Affiliations:** 1 School of Animal Sciences, Virginia Tech, Blacksburg, VA 24060, USA; 2 Division of Animal and Nutritional Sciences, West Virginia University, Morgantown, WV 26506, USA; 3 Prestage Department of Poultry Science, North Carolina State University, Raleigh, NC 27607, USA

**Keywords:** chicken, SRBC, antibody, spleen, bursa of Fabricius

## Abstract

**Simple Summary:**

Lines of White Leghorn chickens divergently selected for high or low antibody response to sheep red blood cells (SRBC) have undergone selection for 50 generations. These lines have allowed for insights into understanding the phenotypic and genotypic changes occurring under phenotypic selection for this single trait. When collecting organ samples from 18-day embryos of generation 48, a difference was seen in the size of the spleen. This observation led to the question of whether the growth of organs associated with immunity had changed after almost 50 generations of selection. Body weights and weights of immune organs representing both primary and secondary immune systems were taken at nine time points. The results showed that divergent selection for the response to SRBC and relaxation of selection resulted in differences in spleen and bursa weights during the later stages of embryonic development and early post-hatch.

**Abstract:**

Long-term divergent selection from a common founder population for a single trait—antibody response to sheep erythrocytes 5 days post-injection—has resulted in two distinct lines of White Leghorn chickens with a well-documented difference in antibody titers: high (HAS)- and low (LAS)-antibody selected lines. Subpopulations—high (HAR)- and low (LAR)-antibody relaxed—were developed from generation 24 of the selected lines to relax selection. The objective of the current experiment was to determine if this long-term selection and relaxation of selection impacted the growth of two organs important to chicken immunity: the spleen and the bursa of Fabricius. Spleens and bursae were obtained from ten chickens per line at nine timepoints (E18, D0, D6, D13, D20, D35, D49, D63, and D91) throughout their rapid growth phase and presented as a percent of body weight. Significance was set at *p* ≤ 0.05. For the spleen, all lines consistently increased in size relative to body weight to D49, followed by a consistent decline. All lines had a similar growth pattern, but HAS spleens grew faster than LAS spleens. For the bursa, LAS was smaller than the other three lines as an embryo and also smaller than HAS through D63. In the selected lines, bursa weight peaked at D35, whereas the relaxed lines peaked at D49. By D91, there was no difference between lines. Artificial and natural selection, represented by the long-term selected and relaxed antibody lines, resulted in differences in the growth patterns and relative weights of the spleen and bursa of Fabricius.

## 1. Introduction

Long-term divergent selection from a common founder population for a single trait—antibody response to sheep erythrocytes 5 days post-injection—has resulted in two distinct lines of White Leghorn chickens with a well-documented difference in antibody titers. These lines were developed for “determining if general fitness could be studied by measuring the genetic variation in response to natural antigens” [1]. Sheep red blood cells (SRBC) are a preferred T-cell-dependent antigen for eliciting a nonpathogenic immune response [2]. At generation 49, the high (HAS)- and low (LAS)-antibody selected lines differed by approximately seven-fold. Subpopulations (HAR and LAR) were developed from generation 24 of the selected lines to relax selection, representing natural selection, instead of the intense artificial selection for HAS and LAS. The HAS and LAS lines differ in their reproductive traits, body weight, and feed efficiency as well as in their resistance to infectious diseases, including *Mycoplasma gallisepticum*, *Escherichia coli*, *Staphylococcus aureus*, *Eimeria tenella*, Newcastle Disease, and Marek’s Disease [3,4,5,6]. These genetic lines have also proven to be a model for genotypic and phenotypic studies to further the understanding of the intestinal microbiome, the MHC-B and MHC-Y complexes, the Usutu virus, and inflammation response to *Histomonas meleagridis* [7,8,9,10,11,12]. However, the effect of selection for high and low antibody response on growth and morphology, particularly of the immune organs, has not been recently analyzed.

The spleen, thought to have originated from the mesoderm, is a secondary immune organ, along with the cecal tonsils. The role of the avian spleen is somewhat different than that of mammals, as it is the primary organ responsible for systemic immune response because of the lack of lymph nodes in poultry. Chicken spleen size has historically been thought to be a possible indicator of health or the presence of disease [13,14,15,16].

The bursa of Fabricius, the thymus, and bone marrow are primary immune organs in chickens. Nicknamed the “cloacal thymus”, the bursa is a dorsal diverticulum located at the junction of the cloaca and large intestine [17]. The bursa is unique to avian anatomy, originating from ectodermal tissue in the developing embryo, and is primarily responsible for B-cell maturation [15,17,18,19].

The spleen and bursa of Fabricius represent both primary and secondary immune systems of poultry. In a preliminary study of generation 48 HAS and LAS chickens, there appeared to be a size difference in the spleen and bursa of Fabricius between lines in 18 d embryos. To further investigate this observation, chickens from the next generation (S49, selected; R26, relaxed) were used to measure their early growth and to obtain spleen and bursa weights relative to body weight, the objective being to determine if long-term selection for high and low antibody response to sheep red blood cells (SRBC) and the relaxation of selection impacted the growth of these two organs, which are important to the chicken immune system.

## 2. Materials and Methods

### 2.1. Animals and Husbandry

The HAS and LAS lines originated from the same founder population of Cornell Randombred White Leghorns and have undergone long-term divergent selection for antibody response to SRBC [6]. Generation 49 HAS and LAS chicks from breeders 40–50 weeks of age (hatch 2 of the main hatch for reproducing the lines), and their respective relaxed lines were incubated under standard conditions. As this experiment aimed to study the long-term impact of selection and the relaxation of selection on bursa and spleen weights in the aforementioned lines of chickens, no SRBC challenge was given to these chicks.

At hatch, the chicks were wing-banded, vaccinated for Marek’s Disease, and placed, by line, in adjacent floor pens with hot-air brooding and concrete floors covered with pine wood shavings. Antibiotic-free feed in mash form (20% CP, 2685 kcal ME/kg) and water were allowed ad libitum. The husbandry and diet were consistent throughout all generations of selection.

At nine timepoints from embryonic day 18 (E18) through day 91 post-hatch (D0, D6, D13, D20, D35, D49, D63, D91), ten chicks per line were weighed and then euthanized by cervical dislocation. Sex was determined by gonadal inspection, and yolk sac, spleen, and bursa weights were obtained. For E18, the yolk sac weight was added to the body weight, because at subsequent sampling ages the yolk sac is drawn into the body cavity and gradually absorbed.

### 2.2. Statistical Analyses

Body weight means are presented, and because means and variances are correlated, they were analyzed using the log_10_ transformation. The spleen and bursa are presented as a percent of body weight, transformed into arc sines of the square root for normality before analysis. All parameters were analyzed by ANOVA [20] to test the main fixed effects and interactions of line, sex, and age at sampling. Significance was considered at *p* ≤ 0.05.

## 3. Results and Discussion

### 3.1. Antibody Response

**Figure 1:**
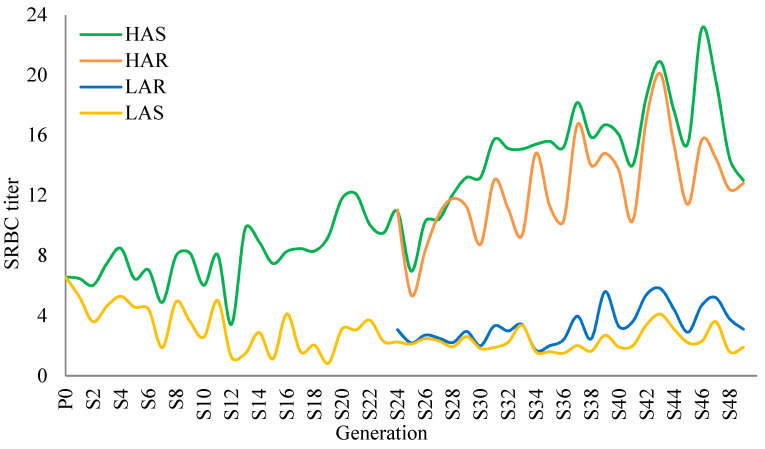
Forty-nine generations of sheep red blood cell (SRBC) antibody titer means for female high (HAS)- and low (LAS)-antibody selected and current high (HAR)- and low (LAR)-antibody relaxed lines.

The divergence of the lines selected for over 49 generations for high or low antibody response to SRBC, as well as where selection was relaxed beginning at generation 24, are shown in Figure 1 (for females). The variation within each line from year to year can partially be explained by the fact that blood has been drawn from a different sheep from the Virginia Tech flock each year. The relaxed lines, while following the same pattern as the selected lines, do not go to the extremes, illustrating that perhaps natural selection brings these lines toward their optimum. “But Natural Selection, as we shall hereafter see, is a power incessantly ready for action, and is immeasurably superior to man’s feeble efforts, as the works of Nature are to those of Art”—Charles Darwin [21]. The means and standard deviations for 5-day post-hatch antibody titers of the hatch contemporaries of the chicks used in this experiment were 13.5 ± 3.2, 12.3 ± 4.0, 3.0 ± 1.2, and 1.9 ± 1.1 for HAS, HAR, LAR, and LAS, respectively. Thirty-eight generations prior, the values were 6.9 ± 0.3 and 3.8 ± 0.2 for HAS and LAS, with sexes pooled [22].

### 3.2. Body Weight

**Figure 2:**
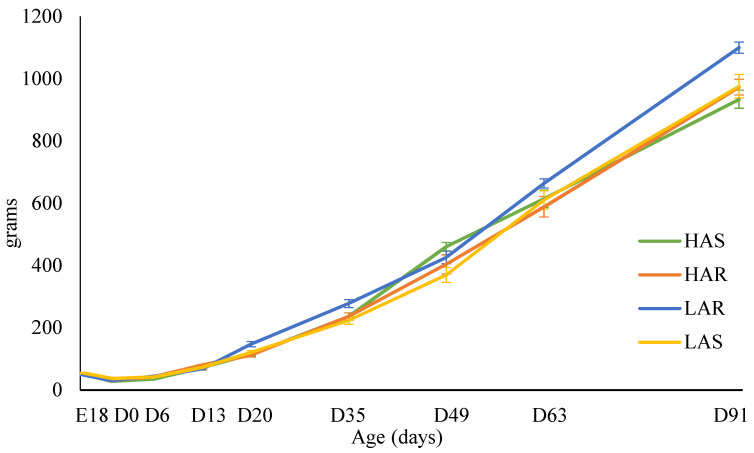
Means (grams) and standard errors for male body weight at each sampling day for high (HAS)- and low (LAS)-antibody selected and high (HAR)- and low (LAR)-antibody relaxed lines.

**Figure 3:**
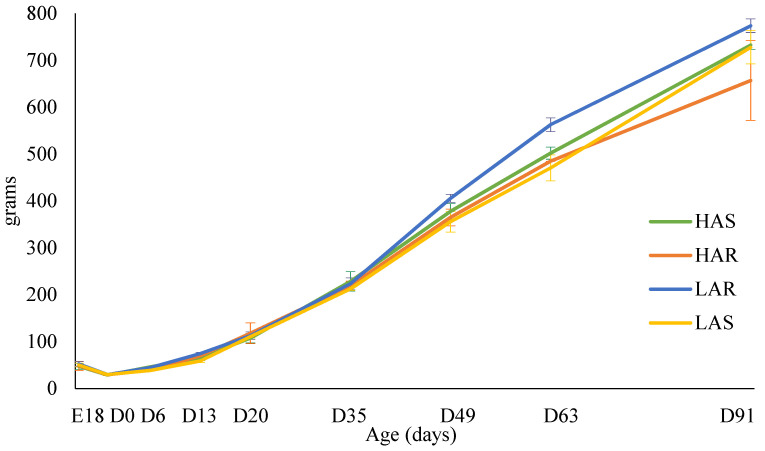
Means (grams) and standard errors for female body weight at each sampling day for high (HAS)- and low (LAS)-antibody selected and high (HAR)- and low (LAR)-antibody relaxed lines.

The mean male and female body weight growth curves of the chicks sampled are shown in Figure 2 and Figure 3, respectively. For males, body weights were not different at E18, D13, or D63. During the initial period of rapid growth (D20 and D35), and again as growth was beginning to slow (D91), LAR males were significantly heavier than the other three lines. Females showed no body weight differences among lines during embryonic development, at hatch, or during the rapid growth period. Natural selection and resource allocation may play a role in allowing the LAR chicks to have a slight increase in growth.

### 3.3. Organ Weight—Body Weight Relationship

There were no significant three-way interactions for line, sex, and age at sampling. However, because males grow at a faster rate to a heavier weight than females and there are differences in weights between lines, one cannot fairly compare absolute organ weights. Ubosi et al. [22] found no sexual dimorphism when adjusting for body weight. Therefore, organ weights were analyzed and presented as a percent of body weight, with sexes pooled.

### 3.4. Spleen Weight, as a Percent of Body Weight

Spleen weights, as a percent of body weight, from E18 to D91 are presented in Table 1. The HAS chicks consistently had greater spleen weights than their relaxed counterparts. The spleens of all four lines had a similar growth pattern, and throughout the trial, both HAS and HAR had larger spleens with respect to body weight than LAR and LAS, with no consistent differences between the LAR and LAS. There was no consistent difference between the two LA lines, but LAR did have larger spleens than LAS with respect to their body weight at D0, D20, and D91. The pattern of immune organ growth and development appears to be a predictor of the functional capabilities of the avian immune system and its responses to antigens and stressors throughout life [23]. At hatch and during the periods just before and after the most rapid growth phases, perhaps natural selection becomes more involved to facilitate the size and function of the spleen. However, to our knowledge, there are no confirmations that a larger spleen, relative to body weight, supports an elevated humoral immunological response to the antigenic properties of SRBC. When an earlier generation of these lines of chickens was fed an aflatoxin, no consistent pattern existed to explain the interaction between relative spleen weight and aflatoxin level [22]. It has been reported that the immune system develops until approximately 34 days of age [15]. For the White Leghorns in the current study, the percent spleen weights among all lines consistently increased until D49, representing the morphological peak time in post-hatch development at 0.279, 0.188, 0.160, and 0.140 percent of body weight for HAS, HAR, LAR, and LAS, respectively. Thereafter, the spleen did not show positive growth relative to body weight.

Relative spleen weights followed the same pattern as the SRBC antibody titers for the four lines—HAS being the largest and LAS being the smallest—with the relaxed lines intermediate yet still significantly different from the other lines. These spleen size differences suggest that the entire long-term single-trait selection experiment has altered the mechanisms used by the lines to organize their resources. Nolin et al. [24] observed differences in the intestinal gene expression of these lines, which also were not injected with SRBC. The HAS line may be prepared to respond to an antibody exposure, while the biological resources of LAS appear to have been redirected toward other physiological fitness traits [23]. Natural selection makes the relaxed lines intermediate, and thus a more balanced approach is required for the allocation of resources as the chicken grows and responds to biological and environmental factors.

### 3.5. Bursa of Fabricius Weight, as a Percent of Body Weight

Important to chicken immunity, the bursa of Fabricius grows rapidly for the first 56–112 days post-hatch, at which time involution begins [18,25,26]. Bursae weights, as a percent of body weight, are presented in Table 2. In the later part of embryonic development, LAS bursae were smaller than in the other lines. By D0, the HAS mean relative bursa weight was significantly greater than LAS, with the relaxed lines being intermediate and not different from either selected line. At D6, the LAS bursa weight, as a percentage of body weight, was again less than the other lines. Throughout the rapid growth period, LAS weight fluctuated between being significantly different and not. The selected-line bursae weights, as a percent of body weight, peaked at D35 (0.428 and 0.281%), and the relaxed lines peaked a week later at 0.358 and 0.310%. By D91, well after the period of the most rapid bursal growth, there were no differences among the lines, with relative bursae weights being 0.232, 0.221, 0.219, and 0.186% for HAS, HAR, LAR, and LAS, respectively.

In a White Leghorn population different from that in the current experiment, bursal growth exceeded the growth of the body only until D21, peaking at 0.52 and 0.50% for males and females, respectively, with a relative bursa weight of 0.14% for females at D49 [17,18]. Subsequently, Glick and Dreesen [26] reported that bursal regression, in two lines selected for large or small bursae, began at 35–56 days of age. Ubosi et al. [22] reported that in a previous generation of the current lines, LAS bursae were smaller than HAS bursae and noted the reciprocal crosses of the two lines. Perhaps the continued presence of antigens in the bursa delays involution [23]. Accordingly, it is also possible that selection for a humoral response to SRBC in these lines of White Leghorns, naïve to this antigen, also caused persistence in relative bursa weight.

The relationship between the size of the bursa and antibody titers is not yet clear. With the growth patterns shown in the present study and in previous work, it appears that the significant difference in bursa weight at hatch may be important to differences in antibody production at older ages [25]. In theory, large bursae, because they have been found to contain greater follicular development at hatch, should be more functional [27]. However, Yamamato and Glick [28] reported an increased antibody response to SRBC in their line selected for small bursa size. Glick and Dreesen [26] found no difference in antibody response to bovine serum albumin between chickens selected for large or small bursae. Thus, weight alone does not determine bursal activity, but rather, what matters is, as Glick described, “the presence of a minimal number of functional cells expressed as lymphocytes within bursal follicles” [27].

### 3.6. The Interface

It appears that selection for high or low antibody titers to SRBC for 49 generations resulted in changes in the weight of the spleen and bursa of Fabricius. There is an interface between the primary and secondary systems; however, it does not appear to be dependent, considering the observation that bursa size did not influence spleen size [25]. As these long-term selected and relaxed lines of chickens approach sexual maturity, resource allocation may be coming into play, with the body making the switch from focusing on the rapid development of the immune system to one of growth and reproduction. This is evidenced by LAS chickens typically having a heavier juvenile body weight, maturing at an earlier age, and having a higher hen-day egg production and percent normal eggs than HAS chickens [6,29]. It is probable that along with growth, the relationships between the spleen and bursa of Fabricius and other systems in the body, such as the intestinal microbiota, work to shape the immune response potential of the chicken [11,22]. These fundamental data provide insight for future genomic and phenotypic immune response studies.

## 4. Conclusions

Differences were observed in the relative weights of the spleen and bursa of Fabricius during the period ranging from E18 to D91 in chickens divergently selected for 49 generations and when selection was relaxed for 26 generations. The developmental growth patterns compared among lines and between organs over time were also different. Thus, long-term artificial and natural selection for immune response to sheep erythrocytes, in chickens from a common founder population, resulted in correlated responses in the relative growth of these two important chicken immune organs—the spleen and bursa of Fabricius—during the critical periods of late embryogenesis and early life post-hatch.

## Figures and Tables

**Table 1:** Means (grams) and standard errors for spleen weight, as a percent of body weight, by age and line (sexes pooled).

Age	HAS	HAR	LAR	LAS
E18	0.035 ± 0.003 ^a^	0.026 ± 0.002 ^b^	0.017 ± 0.001 ^c^	0.015 ± 0.001 ^c^
D0	0.070 ± 0.004 ^a^	0.055 ± 0.003 ^b^	0.042 ± 0.002 ^c^	0.030 ± 0.002 ^d^
D6	0.105 ± 0.011 ^a^	0.093 ± 0.010 ^a^	0.053 ± 0.002 ^b^	0.054 ± 0.003 ^b^
D13	0.172 ± 0.017 ^a^	0.138 ± 0.008 ^b^	0.078 ± 0.005 ^c^	0.062 ± 0.002 ^c^
D20	0.214 ± 0.013 ^a^	0.131 ± 0.010 ^b^	0.095 ± 0.007 ^c^	0.062 ± 0.004 ^d^
D35	0.266 ± 0.013 ^a^	0.160 ± 0.011 ^b^	0.104 ± 0.005 ^c^	0.099 ± 0.009 ^c^
D49	0.279 ± 0.014 ^a^	0.188 ± 0.010 ^b^	0.160 ± 0.009 ^c^	0.140 ± 0.007 ^c^
D63	0.236 ± 0.010 ^a^	0.150 ± 0.011 ^b^	0.110 ± 0.008 ^c^	0.110 ± 0.008 ^c^
D91	0.208 ± 0.011 ^a^	0.148 ± 0.004 ^b^	0.119 ± 0.009 ^c^	0.096 ± 0.006 ^d^

HAS = high antibody selected; HAR = high antibody relaxed; LAR = low antibody relaxed; LAS = low antibody selected; ^a,b,c,d^ Means within a row with uncommon superscripts differ significantly (*p* ≤ 0.05), according to Duncan’s Multiple Range Test; n = 10/line/day.

**Table 2:** Means (grams) and standard errors for bursa of Fabricius weight, as a percent of body weight, by age and line (sexes pooled).

Age	HAS	HAR	LAR	LAS
E18	0.052 ± 0.007 ^a^	0.049 ± 0.003 ^a^	0.051 ± 0.004 ^a^	0.034 ± 0.003 ^b^
D0	0.110 ± 0.009 ^a^	0.092 ± 0.008 ^ab^	0.102 ± 0.005 ^ab^	0.083 ± 0.008 ^b^
D6	0.184 ± 0.015 ^a^	0.156 ± 0.008 ^a^	0.164 ± 0.007 ^a^	0.128 ± 0.011 ^b^
D13	0.281 ± 0.026 ^a^	0.274 ± 0.016 ^a^	0.231 ± 0.011 ^ab^	0.214 ± 0.020 ^b^
D20	0.361 ± 0.021 ^a^	0.286 ± 0.036 ^b^	0.285 ± 0.019 ^b^	0.260 ± 0.012 ^b^
D35	0.428 ± 0.022 ^a^	0.338 ± 0.016 ^b^	0.306 ± 0.021 ^bc^	0.281 ± 0.014 ^c^
D49	0.341 ± 0.016 ^ab^	0.358 ± 0.013 ^a^	0.310 ± 0.013 ^b^	0.261 ± 0.018 ^c^
D63	0.321 ± 0.015 ^a^	0.288 ± 0.014 ^ab^	0.268 ± 0.011 ^bc^	0.247 ± 0.012 ^c^
D91	0.232 ± 0.026	0.221 ± 0.017	0.219 ± 0.008	0.186 ± 0.011

HAS = high antibody selected; HAR = high antibody relaxed; LAR = low antibody relaxed; LAS = low antibody selected; ^a,b,c^ Means within a row with uncommon superscripts differ significantly (*p* ≤ 0.05), according to Duncan’s Multiple Range Test. n = 10/line/day.

## Data Availability

Data are contained within the article.
